# Microalbuminuria in relation to the metabolic syndrome and its components in a Chinese population

**DOI:** 10.1186/1758-5996-3-6

**Published:** 2011-04-07

**Authors:** Chang-Sheng Sheng, Bang-Chuan Hu, Wang-Xiang Fan, Jun Zou, Yan Li, Ji-Guang Wang

**Affiliations:** 1 Centre for Epidemiological Studies and Clinical Trials, Ruijin Hospital, The Shanghai Institute of Hypertension, Shanghai Jiaotong University School of Medicine, Shanghai, China

## Abstract

**Background:**

We investigated the prevalence of microalbuminuria and its association with the metabolic syndrome and its components in a Chinese population.

**Methods:**

The study subjects were recruited from a newly established residential area in the suburb of Shanghai. We measured anthropometry, blood pressure (BP), fasting plasma glucose, and serum lipids, and collected spot urine samples for the determination of albumin-creatinine ratio. We defined microalbuminuria as a urinary albumin-to-creatinine ratio of 30 to 299 mg/g. The metabolic syndrome was defined according to the International Diabetes Federation criteria.

**Results:**

The 1079 participants included 410 (38.0%) hypertensive patients, and 66 (6.1%) diabetic patients. The prevalence of microalbuminuria (4.3%) was 3.2 times higher in 167 patients with the metabolic syndrome than 912 subjects without the metabolic syndrome (12.0% *vs*. 2.9%, *P *< 0.0001). In multiple regression adjusted for sex, age, body mass index, current smoking, alcohol intake and the use of antihypertensive drugs, and mutually adjusted for the components, microalbuminuria was significantly associated with diastolic BP (odds ratio 1.74 for +10 mmHg; 95% confidence interval [CI] 1.10-2.76; *P *= 0.02) and fasting plasma glucose (1.18; 95% CI 1.01-1.41; *P *= 0.04), but not with waist circumference, systolic BP, or serum HDL cholesterol and triglycerides (*P *> 0.10).

**Conclusions:**

Microalbuminuria is common in the Chinese population, and much more prevalent in the presence of the metabolic syndrome, mainly attributable to elevated diastolic BP and plasma glucose.

## Background

Micoralbuminuria is an early marker of chronic kidney disease (CKD) [1] and vascular dysfunction [2], and is associated with a higher risk of renal function loss [1], cardiovascular events [1,3], and all-cause mortality [3]. Microalbuminuria is relatively common in patients with metabolic disorders, such as type 2 diabetes mellitus [4], and has been incorporated into the definition of the metabolic syndrome of the World Health Organization [5]. However, whether microalbuminuria should be an essential component of the metabolic syndrome remains controversial. Indeed, the Adult Treatment Panel III criteria (ATP III) [6], the American Heart Association criteria [7], and the recent harmonized international criteria [8] for the definition of the metabolic syndrome included blood pressure, waist circumference, fasting plasma glucose, serum triglycerides, and high-density lipoprotein (HDL) cholesterol, but excluded microalbuminuria [9].

Nonetheless, several recent studies investigated the relationship of microalbuminuria with the metabolic syndrome and its components [10-14]. In the Third National Health and Nutrition Examination Survey (NHANES III), microalbuminuria was associated with the metabolic syndrome, and mainly with the fasting plasma glucose and blood pressure components [10]. The authors therefore proposed that microalbuminuria should become a component of the metabolic syndrome [10]. Several studies in Eastern Asians confirmed the association between microalbuminuria and the metabolic syndrome, but produced inconsistent results on the association between microalbuminuria and the metabolic syndrome components [11-14]. We therefore performed the present cross-sectional analysis on the association of microalbuminuria with the metabolic syndrome and its components in a Chinese general population sample.

## Methods

### Study population

The present cross-sectional analysis was based on the recent follow-up data of an ongoing longitudinal population study on multiple cardiovascular risk factors in Shanghai. The Ethics Committee of Ruijin Hospital, Shanghai Jiaotong University School of Medicine approved the study protocol. All subjects gave written informed consent. The study subjects were recruited from a newly established residential area in the suburb of Shanghai, 30 kilometers from the city centre. Most residents were immigrants from the nearby villages since 2003, and previously doing farming or other agricultural work. In the period from 2004 to 2006, we invited all residents at least 12 years of age to take part. Of the 2275 invited, 1700 (74.7%) participated in the study.

At the time of the follow-up study in 2009, 1630 of the 1700 subjects were still alive, 1192 (73.1%) participated in the follow-up examinations. We excluded 98 subjects, because they did not have blood (n = 22) or spot urine sampling (n = 58), or anthropometric measurements (n = 18). For the purpose of the present study on microalbuminuria, we also excluded 15 subjects with macroalbuminuria (urinary albumin-to-creatinine ratio ≥ 300 mg/g). Thus, the total number of subjects included in the present analysis was 1079.

### Filed work

One experienced physician measured each participant's blood pressure five times consecutively by mercury sphygmomanometry, after the subjects had rested for at least 5 minutes in the sitting position. These five blood pressure readings were averaged for analysis. The same observer also administered a standardized questionnaire to collect information on medical history, smoking habits, alcohol consumption and the use of medications. Hypertension was defined as a sitting blood pressure of at least 140 mmHg systolic or 90 mmHg diastolic, or as the use of antihypertensive drugs. One trained technician performed anthropometric measurements, including body height, body weight, and waist and hip circumferences. Waist circumference was measured at the smallest circumference between the ribs and iliac crest, and hip circumference at maximum circumference between the iliac crest and crotch to the nearest 0.1 cm.

Venous blood samples were drawn after overnight fasting for the measurement of plasma glucose concentration and for measurements of serum concentrations of total and HDL cholesterol, and triglycerides. Diabetes mellitus was defined as a plasma glucose of at least 7.0 mmol/L fasting or 11.1 mmol/L at any time, or as the use of antidiabetic agents [5]. Fresh spot urine samples were collected for the measurement of urinary albumin and creatine, using immunoturbidimetry and enzymatic methods, respectively. Urinary albumin excretion was expressed as the albumin-to-creatinine ratio (mg/g).

### Definition of microalbuminuria and the metabolic syndrome

We defined microalbuminuria as a urinary albumin-to-creatinine ratio of 30 to 299 mg/g [5], and the metabolic syndrome according to the IDF criteria [8]. The metabolic syndrome could be diagnosed, if in the presence of central obesity (waist circumference ≥ 90 cm for men and ≥ 80 cm for women), plus any two of the following four factors: 1) serum triglycerides ≥150 mg/dL (1.7 mmol/L); 2) serum HDL cholesterol <40 mg/dL (1 mmol/L) in men and <50 mg/dL (1.29 mmol/L) in women; 3) blood pressure ≥130/85 mm Hg or the use of antihypertensive drugs; and 4) fasting plasma glucose ≥100 mg/dL (5.6 mmol/L) or diabetes mellitus.

### Statistical methods

For database management and statistical analysis, we used SAS software (version 9.13, SAS Institute, Cary, NC). Means and proportions were compared with the normal z-test and Fisher's exact test, respectively. Continuous measurements with a skewed distribution were normalized by logarithmic transformation and represented by geometric mean and 95% CI. We performed multiple logistic regression analyses to study the association of microalbuminuria with the metabolic syndrome and its components, while controlling for covariates including sex, age, body mass index, current smoking, alcohol intake and the use of antihypertensive drugs. In further regression analyses, the metabolic syndrome components were also mutually adjusted.

## Results

1079 participants included 412 (38.2%) men and 410 (38.0%) hypertensive patients, of whom 331 (30.7%) took antihypertensive drugs. Men and women had similar systolic blood pressure (mean ± SD; 125.1 ± 18.7 mmHg), pulse rate (68.5 ± 8.4 beats/minute), fasting plasma glucose (4.55 ± 1.34 mmol/L), serum total cholesterol (4.74 ± 0.90 mmol/L), and prevalence of diabetes mellitus (6.1%, Table 1). Men, compared with women, were slightly older (+2 years, *P *= 0.01), had a significantly (*P *≤ 0.05) greater body mass index (+0.5 kg/m^2^), higher diastolic blood pressure (+3.4 mmHg), and lower HDL cholesterol (-0.19 mmol/L), and reported higher (*P *< 0.0001) proportions of current smoking (47.8% *vs*. 0.5%) and alcohol intake (35.4% *vs*. 1.2%). Overall, 20 (1.9%) subjects reported a history of stroke, and 1 (0.1%) had coronary artery disease.

**Table 1:** Characteristics of the study population

Characteristic	Men (n = 412)	Women (n = 667)	*P *value
Demography			
Age, years	57.0 ± 12.7	55.0 ± 12.9	0.01
Body mass index, kg/m^2^	24.2 ± 3.5	23.7 ± 3.2	0.02
Waist-to-hip ratio	0.89 ± 0.06	0.86 ± 0.32	0.04
Blood pressure, mm Hg			
Systolic	125.9 ± 17.5	124.7 ± 19.4	0.28
Diastolic	78.8 ± 9.6	75.4 ± 9.1	<0.0001
Pulse rate, beats/minute	68.1 ± 8.9	68.7 ± 8.0	0.29
Take antihypertensive drugs, n (%)	139 (33.8)	192 (28.8)	0.09
Hypertension, n (%)^a^	179 (43.6)	231 (34.7)	0.004
Diabetes mellitus, n (%)^a^	28 (6.8)	38 (5.7)	0.41
Biochemical measurements			
Plasma fasting glucose, mmol/L	4.50 ± 1.55	4.57 ± 1.19	0.42
Serum total cholesterol, mmol/L	4.73 ± 0.87	4.74 ± 0.91	0.90
Serum HDL cholesterol, mmol/L	1.62 ± 0.45	1.81 ± 0.41	<0.0001
Serum triglycerides, mmol/L	1.53 (1.44-1.63)	1.27 (1.22-1.33)	<0.0001
Microalbuminuria, n (%)^a^	**14 (3.4)**	**32 (4.8)**	**0.26**
Metabolic syndrome, n (%)^a^	**60 (14.6)**	**107 (16.0)**	**0.52**

Values are mean ± SD, geometric means (95% confidence interval), or the numbers of subjects (%).

HDL, high density lipoprotein.

^a^For definitions of hypertension, diabetes mellitus, microalbuminuria and the metabolic syndrome, see Methods.

**Figure 1:**
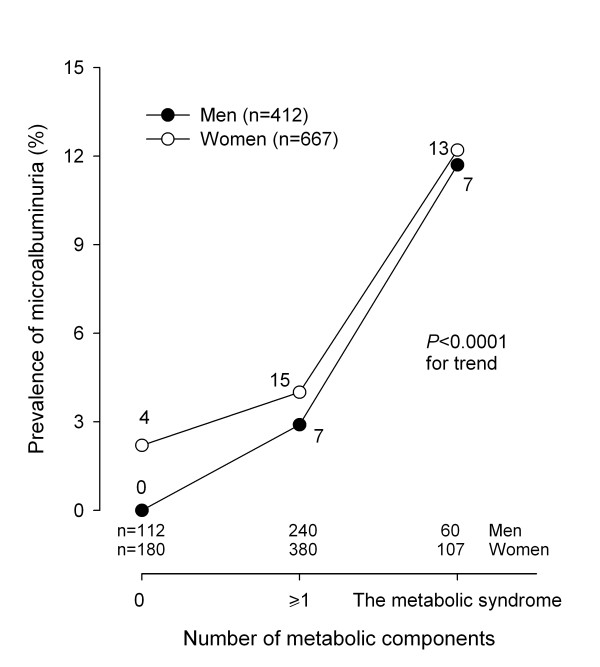
**The prevalence of microalbuminuria by number of the metabolic syndrome components subjects in men (closed circles) and women (open circles) separately**. On the horizontal axis, 0 and ≥1 indicate subjects without any metabolic syndrome components and those with at least 1 component but not diagnosed as the metabolic syndrome, respectively. The number of subjects per subgroup is given at the bottom, and those with microalbuminuria alongside the symbols. *P *< 0.0001 for trend in men and women combined.

Men and women had similar prevalence of microalbuminuria (3.4% *vs*.4.8%; *P *= 0.26) and the metabolic syndrome (14.6% *vs*.16.0%; *P *= 0.52). The prevalence of microalbuminuria was higher with increasing number of the metabolic syndrome components (Figure 1). Indeed, the prevalence of microalbuminuria was 1.4% in subjects without any metabolic syndrome components, and increased to 3.6% in subjects with at least 1 metabolic component but not diagnosed as the metabolic syndrome, and to 12.0% in subjects with the metabolic syndrome (*P *< 0.0001 for trend).

In unadjusted continuous analyses, microalbuminuria was significantly (*P *< 0.01) associated with all the metabolic syndrome components, except serum HDL cholesterol (*P *= 0.59, Table 2). After adjustment for sex, age, body mass index, current smoking, alcohol intake and the use of antihypertensive drugs, the association remained statistically significant (*P *< 0.01) for systolic and diastolic blood pressure, and fasting plasma glucose. However, after mutual adjustment for the components of the metabolic syndrome, microalbuminuria was only significantly associated with diastolic blood pressure (odds ratio 1.74 for each 10 mmHg increase; 95% confidence interval [CI] 1.10-2.76; *P *= 0.02) and fasting plasma glucose (1.18 for each 1 mmol/L increase; 95% CI 1.01-1.41; *P *= 0.04).

**Table 2:** Association of microalbuminuria with the metabolic syndrome components as continuous variables

The metabolic syndrome component	Unadjusted		**Adjusted Model 1**^**a**^		**Adjusted Model 2**^**b**^	
	Odds ratio (95% CI)	*P *value	Odds ratio (95% CI)	*P *value	Odds ratio (95% CI)	*P *value
Waist circumference, +10 cm	1.64 (1.19-2.27)	0.003	1.43 (0.78-2.64)	0.25	1.30 (0.69-2.45)	0.42
Systolic blood pressure, +10 mm Hg	1.35 (1.17-1.55)	<0.0001	1.32 (1.11-1.56)	0.002	1.08 (0.85-1.37)	0.55
Diastolic blood pressure, +10 mm Hg	1.90 (1.41-2.50)	<0.0001	1.97 (1.41-2.76)	<0.0001	1.74 (1.10-2.76)	0.02
Fasting plasma glucose, mmol/L	1.24 (1.08-1.42)	0.003	1.22 (1.05-1.42)	0.008	1.18 (1.01-1.41)	0.04
Serum triglycerides, mmol/L	1.10 (1.01-1.22)	0.04	1.09 (0.98-1.21)	0.12	1.03 (0.91-1.17)	0.63
Serum HDL cholesterol, mmol/L	0.83 (0.41-1.66)	0.59	0.94 (0.43-2.04)	0.87	1.30 (0.58-2.96)	0.53

CI, confidence interval; HDL, high density lipoprotein.

^a^Adjusted for sex, age, body mass index, current smoking, alcoholic intake and use of antihypertensive drugs.

^b^Additionally adjusted for the other components of the metabolic syndrome.

The categorical analysis was confirmatory (Table 3). In fully adjusted analyses, the risk of microalbuminuria was 116% higher in patients with the blood pressure component (systolic/diastolic blood pressure ≥130/85 mmHg, *P *= 0.04), mainly because of elevated diastolic blood pressure (odds ratio 2.25; 95% CI 1.09-4.65; *P *= 0.03), and was 157% higher in patients with elevated fasting plasma glucose (*P *= 0.007).

**Table 3:** Association of microalbuminuria with the metabolic syndrome components as dichotomized variables

The metabolic syndrome component	Number (Prevalence, %)	Unadjusted		**Adjusted Model 1**^**a**^		**Adjusted Model 2**^**b**^	
		Odds ratio (95% CI)	*P *value	Odds ratio (95% CI)	*P *value	Odds ratio (95% CI)	*P *value
Central obesity	422 (39.1)	3.38 (1.80-6.34)	0.0001	2.52 (1.09-5.83)	0.03	2.24 (0.96-5.24)	0.06
Elevated blood pressure	522 (48.4)	3.16 (1.62-6.18)	0.0007	2.85 (1.22-6.67)	0.02	2.16 (1.01-4.63)	0.04
Elevated fasting plasma glucose	126 (11.7)	3.53 (1.83-6.81)	0.0002	3.03 (1.55-5.94)	0.001	2.57 (1.29-5.11)	0.007
Elevated serum triglycerides	356 (33.0)	2.10 (1.16-3.80)	0.01	1.75 (0.94-3.26)	0.08	1.30 (0.67-2.55)	0.44
Reduced serum HDL cholesterol	87 (8.1)	2.14 (0.93-4.94)	0.07	1.71 (0.71-4.08)	0.23	1.29 (0.51-3.25)	0.59

CI, confidence interval; HDL, high density lipoprotein.

The metabolic syndrome component was defined according to the Adult Treatment Panel III criteria (ATP III) criteria [6]. For further information, see methods.

^a^Adjusted for sex, age, body mass index, current smoking, current alcoholic intake and use of antihypertensive drugs (except for elevated blood pressure).

^b^Additionally adjusted for the other components of metabolic syndrome.

After exclusion of 331 users of antihypertensive drugs, we performed sensitivity analysis in 748 subjects not taking antihypertensive drugs (Table 4). The number of subjects with microalbuminuria and the metabolic syndrome decreased substantially from 46 to 24 and from 167 to 64, respectively. Nonetheless, with similar adjustments applied as above, microalbuminuria was strongly associated with the metabolic syndrome (odds ratio 5.13, *P *= 0.001).

**Table 4:** Sensitivity analysis on the association between microalbuminuria and the metabolic syndrome or its components

The metabolic syndrome component	Number (Prevalence, %)	Adjusted odds ratio (95% CI)*	*P *value
Central obesity	248 (33.2)	1.53 (0.47-5.03)	0.48
Elevated blood pressure	193 (25.8)	2.20 (0.90-5.40)	0.08
Elevated fasting plasma glucose	66 (8.8)	2.30 (0.83-6.42)	0.11
Elevated serum triglycerides	210 (28.1)	1.65 (0.66-4.12)	0.48
Reduced serum HDL cholesterol	52 (7.0)	1.88 (0.54-6.50)	0.32
The metabolic syndrome	64 (8.6)	5.13 (1.96-13.45)	0.001

CI, confidence interval; HDL, high density lipoprotein.

* Adjusted for sex, age, body mass index, current smoking, current alcoholic intake and use of antihypertensive drugs (except for analyses on elevated blood pressure and in subjects not taking antihypertensive medication) and additionally for the other components of the metabolic syndrome (except for analyses on the metabolic syndrome).

## Discussion

In our population-based study, we found that microalbuminuria was common in the Chinese population (4.3%) in general and much more prevalent in persons with the metabolic syndrome (12.0%) in particular. Among the 5 commonly considered components of the metabolic syndrome, blood pressure and plasma glucose were the major determinants of microalbuminuria.

Although we used lower sex-specific cut-off limits to define microalbuminuria, the prevalence of microalbuminuria in our study population was slightly lower than several previous population studies in Chinese [11,13,14], Japanese [12], or US Americans [10], in which the similar urinary albumin-to-creatinine ratio technique was used in the assessment of microalbuminuria. Indeed, the prevalence of microalbuminuria was 6.7% in 3532 Chinese (mean age 50 years) living in the urban area of Shanghai [11], 8.8% in 2985 Chinese (mean age 44 years) living in a city 200 km south of Shanghai [14], 11.5% in 2311 Chinese living in Taiwan (mean age 57 years) [13], 13.7% in 2321 Japanese (mean age 60 years) [12], and 6.4% in 5659 participants of the NHANES study (20-80 years) [10]. The discrepancy in the prevalence of microalbuminuria across these populations might be attributable to the differences in the characteristics of study participants, such as age and cardiovascular risk factors.

Our finding on the association between microalbuminuria and the metabolic syndrome is in line with the results of the recently published studies [10-14]. The prevalence of microalbuminuria was consistently higher (*P *< 0.0001 for all) in persons with the metabolic syndrome than those without in Chinese (20.3% *vs*. 2.0%) [14], Japanese (20.8% *vs*. 12.2%) [12], and US Americans (13.7% *vs*. 4.8) [10]. With regard to the association with specific metabolic syndrome components, 3 previous studies [10-12] and our research produced similar results. In 3532 Chinese, the risk of microalbuminuria was significantly associated with high blood pressure (odds ratio 2.15; *P *< 0.001) and hyperglycemia (odds ratio 1.64; *P *= 0.01), but not with other components of the metabolic syndrome [11]. In 2321 Japanese, microalbuminuria was significantly associated with elevated blood pressure (odds ratio 2.37; *P *< 0.001) and hyperglycemia (odds ratio 2.64; *P *< 0.001), weakly with central obesity (odds ratio 1.31; *P *= 0.04), but not with other components of the metabolic syndrome [12]. In the NHANES study, microalbuminuria was associated with high blood pressure and hyperglycemia in men (odds ratio 2.51; *P *< 0.001 for both) as well as women (odds ratio 3.34; *P *< 0.001 and 2.24; *P *= 0.0047, respectively), and weakly with central obesity in men (odds ratio 2.05; *P *< 0.02) [10]. However, 2 other studies demonstrated that microalbuminuria was associated with all components of the metabolic syndrome [13,14], probably because in these 2 studies the components of the metabolic syndrome were not mutually adjusted. Nonetheless, as exemplified in the present study, in subjects without established cardiovascular disease, such as treated hypertension might also be a significant correlate of microalbuminuria.

The mechanism for the closer relationship of microalbuminuria with diastolic blood pressure and hyperglycemia is not entirely understood. Microalbuminuria is a marker of endothelial dysfunction and vascular damage [2,15]. The metabolic syndrome is basically a disorder in the metabolism of glucose and lipids [1]. However, recent studies suggest that the metabolic syndrome can also be a disease in arterioles [16]. Peripheral circulation is the major determinant of diastolic blood pressure and glucose disposal. Before type 2 diabetes mellitus or systolic hypertension is developed, the metabolic syndrome is probably a vascular disease in the periphery, which may cause mainly high diastolic blood pressure and hyperglycemia.

Our study should be interpreted within the context of its limitations. First, we only collected a random spot urine sample. Though adjusting for urinary creatinine excretion, the assessment of albumin excretion might be less accurate than 24-hour urine collections or first morning voids [17,18]. However, the spot urine sampling also has advantages, such as rapid deep freezing after collection, in the absence of any antiseptic agent. Second, our study had a relatively small sample size. Only 46 persons had microalbuminuria and 167 persons had the metabolic syndrome. However, this shortfall might be overcome by intensive and accurate measurement of all the variables under study. Third, our study was cross-sectional and hence no causal conclusion could be drawn.

In conclusion, microalbuminuria is common in the Chinese population, and is much more prevalent in persons with the metabolic syndrome, mainly attributable to high diastolic blood pressure and high fasting plasma glucose. Microalbuminuria is currently an indication of interventions, such as blood pressure lowering [2], even in the absence of hypertension or diabetes. However, the metabolic syndrome is still considered as a controversial and ambiguous phenomenon and by and large inadequately managed [19]. One of the important implications of our study is that we probably should consider to screen microalbuminuria in persons with prehypertension [20] or prediabetes [21], such as the blood pressure and glucose components of the metabolic syndrome, and to identify those at high cardiovascular risk but not on any proven effective therapeutic treatment.

## Authors' contributions

CSS participated in the epidemiological and laboratory work, performed statistical analysis, and together with JGW drafted the manuscript. BCH, WXF, and JZ participated in the epidemiological work. YL and JGW conceived of, designed and coordinated the study. All authors read and approved the final manuscript.

## Competing interests

The authors declare that they have no competing interests.
